# Community Health Worker Delivered Pain Self Management Improves Function in Older Adults with Cognitive Impairment

**DOI:** 10.1093/geroni/igaf122.3900

**Published:** 2025-12-31

**Authors:** Jennifer Blackwood, Donovan Maust, Rachel Davis, Elizabeth Brines, Charity Hoffman, Amanda Leggett, Joan Ilardo, Mary Janevic

**Affiliations:** University of Michigan-Flint, Flint, Michigan, United States; University of Michigan Medical School, Ann Arbor, Michigan, United States; University of Michigan, Ann Arbor, Michigan, United States; University of Michigan, Ann Arbor, Michigan, United States; University of Michigan Health System, Ann Arbor, Michigan, United States; Wayne State University, Detroit, Michigan, United States; Michigan State University, East Lansing, Michigan, United States; University of Michigan School of Public Health, Ann Arbor, Michigan, United States

## Abstract

Older adults with chronic pain and mild cognitive impairment or Alzheimer’s disease–related dementias (MCI/ADRD) face dual challenges that undermine function and quality of life, yet few interventions address both conditions. We developed and tested STEPS-CI, a telephone-based pain self-management program delivered by community health workers (CHWs) and adapted for cognitive impairment. Fifty-two participants (mean age 67.1; 83% women; 46% African American; 27% rural, 44% Detroit-based) were randomized to intervention (n = 23) or control (n = 29). Participants in the intervention arm received seven weekly CHW-led sessions, supported by tailored materials co-designed with an advisory council from the National Council of Dementia Minds. At post-program follow-up, Global Impression of Change ratings showed significant improvements in function, pain, and quality of life for intervention vs. control participants (all p <.01) as well as in social participation (PROMIS 4-item scale, p=.03). These gains were accompanied by favorable trends in reduced pain interference and decreased self-reported pain medication use. The program was feasible and well-accepted, with 82% retention, >90% CHW fidelity to session protocols, and strong qualitative feedback highlighting relevance and accessibility. Findings suggest that CHWs can successfully deliver adapted behavioral pain management strategies to older adults with MCI/ADRD, producing functional benefits that matter to patients and families. This pilot study provides new data supporting advancement to a fully powered Behavioral Intervention Stage 3 trial to evaluate the efficacy of STEPS-CI for improving pain-related outcomes in diverse older adults living with both pain and dementia.

